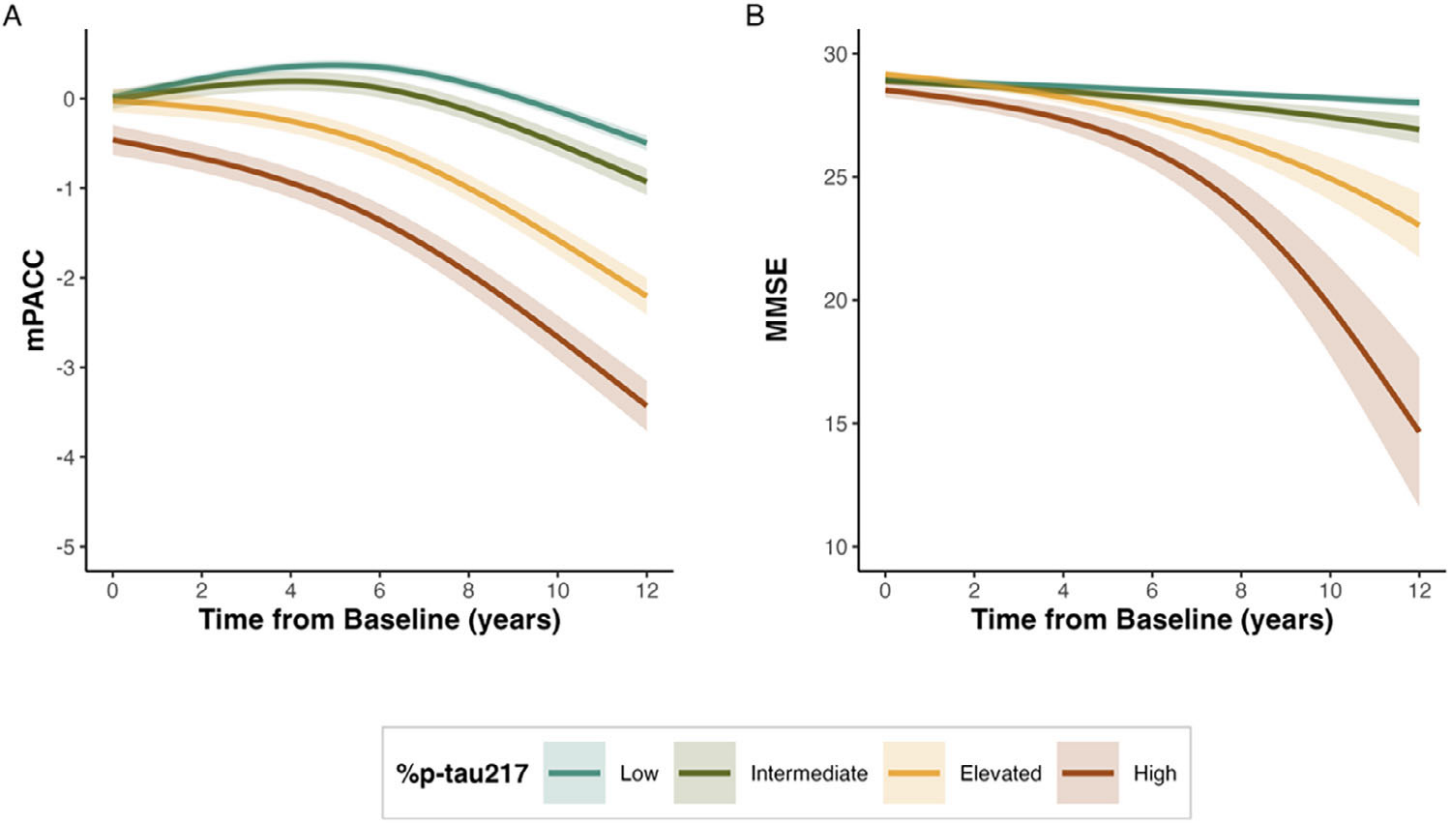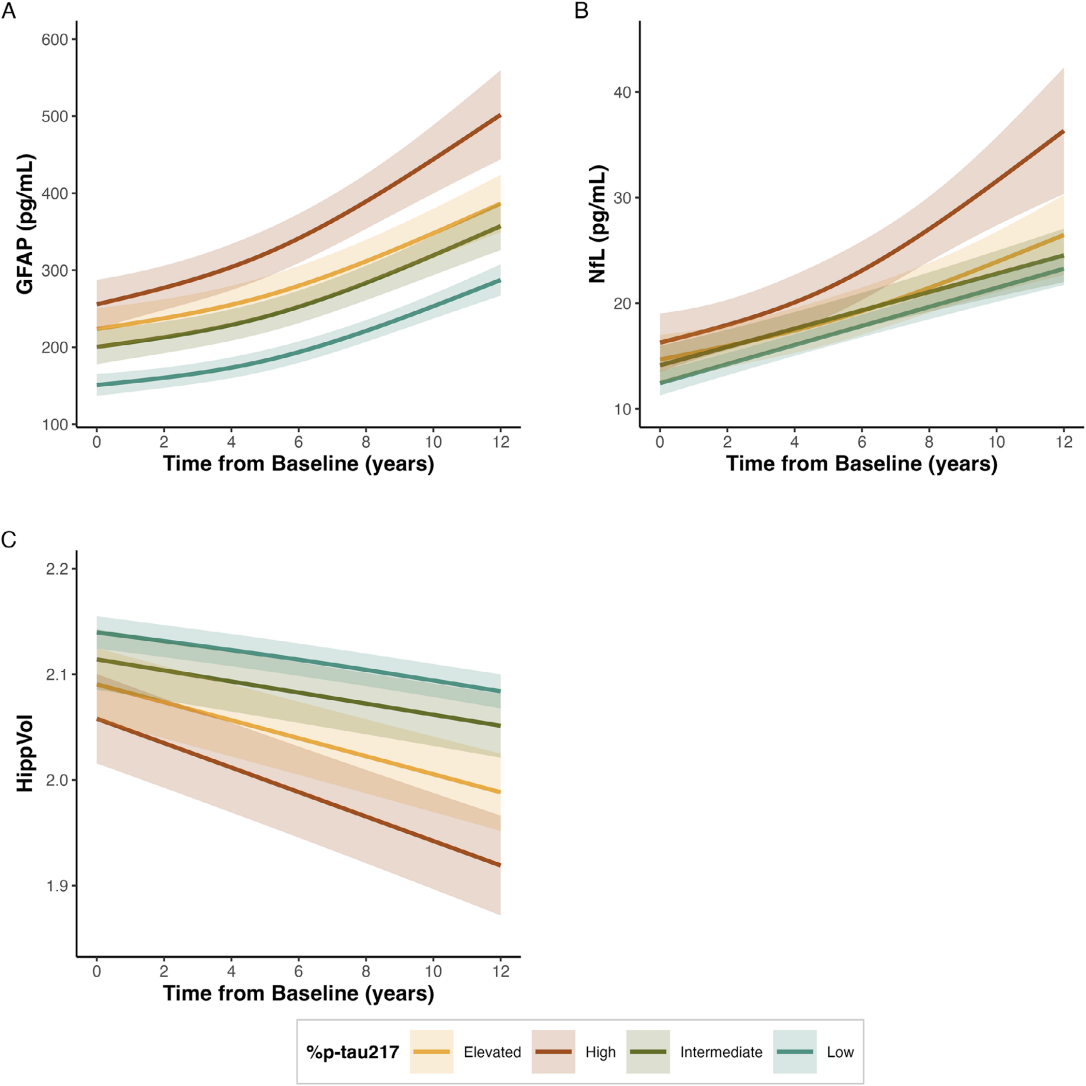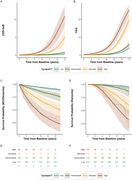# Long‐term trajectories of cognition, neurodegeneration, and clinical progression in cognitively unimpaired individuals with elevated levels of plasma %*p*‐tau217

**DOI:** 10.1002/alz70861_108362

**Published:** 2025-12-23

**Authors:** Jesús Silva‐Rodríguez, Linda Zhang, Luca Kleineidam, Sonia Wagner, Elizabeth Lucia Valeriano‐Lorenzo, Teodoro del Ser, Michel J. Grothe, Pascual Sánchez‐Juan

**Affiliations:** ^1^ CIEN Foundation, Reina Sofia Alzheimer Center, ISCIII, Madrid, Madrid Spain; ^2^ Department of Old Age Psychiatry and Cognitive Disorders, University Hospital Bonn and University of Bonn, Bonn Germany; ^3^ CIEN Foundation, Reina Sofía Alzheimer Centre, ISCIII, Madrid, Madrid Spain; ^4^ Alzheimer’s Centre Reina Sofia Foundation‐CIEN Foundation‐ISCIII, Madrid, Madrid Spain; ^5^ Alzheimer's Center Reina Sofia‐CIEN Foundation, Madrid, Madrid Spain

## Abstract

**Background:**

%*p*‐tau217 ratio has emerged as a promising plasma biomarker for Alzheimer’s disease (AD) pathology, reaching performance levels comparable to CSF‐based measures. However, its prognostic value in cognitively unimpaired (CU) individuals remains to be further explored.

**Method:**

We studied 982 CU older adults (age: 75 ± 4 years; 64% female) from the Vallecas Project (CIEN, Madrid, Spain), with annual follow‐ups over up to 12 years (mean: 7.9 ± 3.3). Baseline plasma %p‐tau217 was quantified using mass spectrometry and used to stratify subjects into four risk categories using a predefined three‐cut‐off model. Longitudinal changes in a) cognition (mPACC, MMSE); b) hippocampal volume; c) plasma biomarkers of astrocytic reactivity/neurodegeneration (GFAP, NfL); and d) clinical/functional measures (CDR‐SB, FAQ) were assessed. Risk of progression to MCI/dementia was evaluated using survival analysis. All models were adjusted for age, sex, and APOE4. Positive and Negative predictive value (PPV, NPV) were estimated through time‐dependent ROC analysis.

**Result:**

Participants were classified as having “Low” (*n* =674, 68.6%), “Intermediate” (*n* =145, 14.8%), “Elevated” (*n* =103, 10.5%), or “High” (*n* =60, 6.1%) %*p*‐tau217 levels. Cognitive decline (mPACC, MMSE) accelerated with increasing %*p*‐tau217 (*p* <0.001, Fig‐1). GFAP showed baseline group differences, but steeper longitudinal increases were restricted to the “High” group (Fig‐2A, *p* <0.001). NfL changes followed similar, though less significant patterns (Fig‐2B). Hippocampal atrophy was significantly faster in both the “Elevated” and “High” groups compared to “Low”/“Intermediate” (Fig‐2C, *p* <0.001), so was clinical (CDR‐SB) and functional (FAQ) decline (Fig‐3, A‐B; *p* <0.001). Survival analysis (Fig‐3, C‐F) showed significantly increased risk of progressing to MCI (χ²=179, *p* <0.001) and dementia (χ²=137, *p* <0.001) in higher %*p*‐tau217 groups, with HR>6 for the “Elevated” and “High” groups. The “Low” group showed strong NPV for MCI conversion even at 10 years (92.1%), while “High” showed moderate PPVs for MCI (55% at 5 years, 71% at 10 years) and dementia (34% and 41%).

**Conclusion:**

Plasma %*p*‐tau217 represents a robust and scalable biomarker for early identification of CU individuals at increased risk of AD‐related neurodegeneration and cognitive decline, reinforcing its potential role in both clinical practice and research trials. Notably, individuals in the lower %*p*‐tau217 categories demonstrated minimal risk of clinical progression even over long‐term follow‐up.